# Donor caveolin 1 (*CAV1*) genetic polymorphism influences graft function after renal transplantation

**DOI:** 10.1186/s13069-015-0025-x

**Published:** 2015-05-05

**Authors:** Cynthia Van der Hauwaert, Grégoire Savary, Claire Pinçon, Viviane Gnemmi, Christian Noël, Franck Broly, Myriam Labalette, Michaël Perrais, Nicolas Pottier, François Glowacki, Christelle Cauffiez

**Affiliations:** EA4483, Département de Biochimie et Biologie Moléculaire, Faculté de Médecine, Pôle Recherche, Université de Lille, 1 place de Verdun, Lille Cedex, 59045 France; Laboratoire de Biomathématiques, Faculté des Sciences Pharmaceutiques, Université de Lille, 3 rue du Professeur Laguesse - BP 83, 59006 Lille Cedex, France; Institut de Pathologie, Centre de Biologie Pathologie Génétique, CHRU, Boulevard du Professeur Jules Leclercq, 59037 Lille Cedex, France; Service de Néphrologie, Hôpital Huriez, CHRU, 2 avenue Oscar Lambret, 59037 Lille Cedex, France; Service d’Immunologie, Centre de Biologie Pathologie Génétique, CHRU, Boulevard du Professeur Jules Leclercq, 59037 Lille Cedex, France; Institut National de la Santé et de la Recherche Médicale, U837, Jean-Pierre Aubert Research Center, Equipe 5 “Mucines, Différenciation et Cancérogenèse Épithéliales”, 1 place de Verdun, 59045 Lille Cedex, France

## Abstract

**Background:**

Identification of the culprit genes underlying multifactorial diseases is one of the most important current challenges of molecular genetics. While recent advances in genomics research have accelerated the discovery of susceptibility genes, much remains to be learned about the functions of disease-associated genetic variants. Recently, Moore and co-workers identified, in the donor genome, an association between a common genetic variant (rs4730751) in the gene encoding caveolin-1 (CAV1), a major structural component of caveolae, and long-term allograft survival.

**Methods:**

Four hundred seventy-five renal recipients consecutively transplanted were included in this study. Donor genomic DNA was extracted and used to genotype *CAV1* rs4730751 Single Nucleotide Polymorphism.

**Results:**

Patients receiving a graft carrying *CAV1* rs4730751 AA genotype displayed a significant decrease in estimated glomerular filtration rate and a significant increase in serum creatinine in both univariate and multivariate analyzes. Moreover, patients receiving a graft with *CAV1* AA genotype significantly developed more interstitial fibrosis lesions on systematic biopsies performed 3 months post-transplantation.

**Conclusions:**

Genotyping of *CAV1* may be relevant to identify patients at risk of adverse renal transplant outcome.

## Background

Most common diseases are complex and result from multiple genetic and environmental factors. The recent advances in genotyping and sequencing technologies have revolutionized our understanding of the genetics of complex traits. For instance, more than 2,600 associated common risk alleles have been identified, with convincing associations in about 350 different complex traits [1]. Nevertheless for the vast majority of associated alleles, the identities of causal genes and variants, as well as their function, remain unclear.

Fibrosis refers to the excessive and persistent formation of scar tissue, which is responsible for morbidity and mortality associated with organ failure in a variety of chronic diseases [2]. Renal fibrosis is a central feature of all progressive renal diseases that ultimately leads to end-stage renal failure. In particular, kidney fibrosis is especially common in renal allografts and is a major cause of allograft dysfunction and loss [3]. This fibrotic process results from numerous injuries related to immune allograft rejection or non immune-mediated chronic damages including calcineurin inhibitor toxicity. Broadly speaking, renal fibrosis is characterized by an excessive wound-healing process of the kidney tissue after chronic, sustained injury resulting in an excessive accumulation of extracellular matrix components. Despite intensive studies, the underlying cause or genetic factors involved in the pathogenesis of renal fibrosis are still largely unknown. Recently, Moore and co-workers identified, in the donor genome, an association between a common genetic variant rs4730751 in the gene encoding caveolin-1 (*CAV1*), a major structural component of caveolae, and long-term allograft survival. More precisely, grafts carrying AA genotype are associated with a higher graft loss frequency (38.6% for AA genotypes *vs* 22.3% and 22.2% for AC and CC genotypes, respectively, at 12 years post-transplant) [4]. Nevertheless, the impact of *CAV1* polymorphism on renal allograft function, or graft histology, is currently unknown.

Caveolin-1 (CAV1), the primary structural component of specialized plasma membrane microdomains called caveolae, is a crucial regulator of tissue fibrosis. Caveolae are involved in numerous biological functions ranging from endocytosis and transcytosis to signal transduction [5,6]. In the kidney, CAV1 is expressed in mesangial cells, epithelial cells, Bowman’s parietal epithelial cells, as well as in renal proximal tubular epithelial cells [7]. As expected from their function and tissue distribution, caveolae and CAV1 are implicated in a variety of human disorders, including cancer and cardiovascular and inflammatory diseases [6,8,9]. In particular, caveolae have a well-described profibrotic role in the context of transforming growth factor beta (TGFβ) signaling. Whereas TGFβ receptor endocytosis *via* clathrin-coated pit-dependent internalization promotes TGFβ signaling, the lipid raft-caveolar internalization pathway facilitates the degradation of TGFβ receptors, therefore decreasing TGFβ signaling [10-12]. In line with this, *CAV1* null mice exhibit an extensive interstitial fibrosis following unilateral ureteral obstruction, a disease model of TGFβ-driven renal fibrogenesis [13].

The aim of this study was to evaluate, in an independent large cohort of transplant recipients receiving a tacrolimus-based immunosuppressive protocol, the impact of donor *CAV1* rs4730751 genetic variant on renal transplant function, graft histology, and graft survival.

## Results

### Description of the cohort

In this retrospective survey, the mean follow-up was 4.8 ± 2.6 years after renal transplantation. *CAV1* rs4730751 AA, AC, and CC genotypes were observed in respectively 7.6% (*n* = 36), 40.8% (*n* = 194), and 51.6% (*n* = 245) of patients. Alleles A and C frequencies (respectively 0.28 and 0.72) are in equilibrium with the Hardy Weinberg law. Demographic and clinical parameters were not significantly different between AA, AC, or CC genotype groups (Table 1).

**Table 1:** **Demographic and clinical characteristics according to** ***CAV1*** **genotype (rs4730751)**

	***CAV1*** **genotype (** ***n*** **= 475)**		
	***AA***	***AC***	***CC***
	***n*** **= 36 (7.6%)**	***n*** **= 194 (40.8%)**	***n*** **= 245 (33.2%)**
BMI (kg/m^2^)	23.4 ± 4.0	24.4 ± 4.6	24.1 ± 4.3
Age at transplantation (years)	44.0 ± 14.4	47.8 ± 12.5	47.5 ± 12.3
Sex of the recipient (M/F)	69.4%/30.6%	66.0%/34.0%	60.0%/40.0%
Age of donor (years)	41.9 ± 15.8	45.8 ± 14.9	45.4 ± 15.1
Sex of the donor (M/F)	68.6%/31.4%	70.3%/29.7%	69.0%/31.0%
Initial nephropathy			
Glomerulonephritis	31.4%	33.3%	32.1%
Interstitial nephropathy	14.3%	17.4%	21.0%
Vascular and diabetic nephropathy	14.3%	14.4%	8.3%
Hereditary nephropathy	17.1%	15.9%	21.0%
Other	8.6%	8.2%	5.6%
Undetermined	14.3%	10.8%	11.9%
Immunization (HLA antibody >30%)	2.8%	10.9%	9.8%
Cold ischemia time (h)	20.8 ± 6.9	21.3 ± 6.9	20.8 ± 6.7
HLA mismatch (A/B/DR)	3.6 ± 1.4	3.4 ± 1.9	3.4 ± 1.3
Delayed graft function	12.9%	25.7%	20.3%
Acute rejection	8.6%	15.6%	9.8%
NODAT	17.3%	15.7%	20.6%
CMV disease	5.6%	10.8%	9.8%
Proteinuria (year 2)	10%	17.3%	19.4%
Proteinuria (year 5)	35.7%	21.3%	13.8%
Anti CD-25 antibody induction	41.4%	44.4%	48.2%
ATG induction	58.6%	55.6%	51.8%
Immunosuppressive therapy (year 1)			
Steroids therapy (%)	40	36	42
Mycophenolate daily dose (g/day)	1.23 ± 0.26	1.40 ± 0.51	1.31 ± 0.60
Tacrolimus daily dose (mg/kg/day)	0.08 ± 0.03	0.08 ± 0.04	0.09 ± 0.04
Tacrolimus trough blood level (ng/mL)	7.36 ± 3.40	8.40 ± 3.29	7.69 ± 3.30

NODAT: new onset diabetes after transplantation, BMI: body mass index, HLA: human leukocyte antigen, ATG: antithymoglobulin.

### Association between rs4730751 and renal graft function

**Figure 1:**
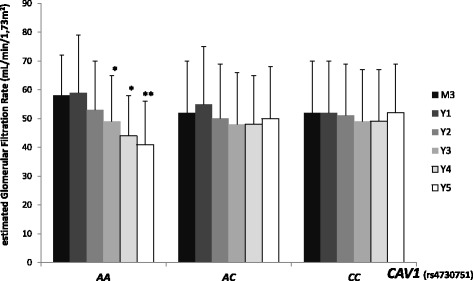
Evolution of the estimated glomerular filtration rate according to donor *CAV1* genotype (rs4730751) between 2 and 5 years post-transplant. Estimated glomerular filtration rate was evaluated according to aMDRD. Data are described as mean ± standard deviation. ^*^ *P* < 0.05 *vs* 2 years ^**^ *P* < 0.005 *vs* 2 years.

**Figure 2:**
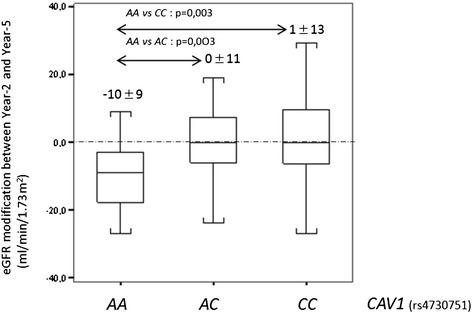
Estimated glomerular filtration rate modification according to donor *CAV1* genotype (rs4730751) between 2 and 5 years post-transplant. Estimated glomerular filtration rate was evaluated according to aMDRD.

Renal function and proteinuria were evaluated according to *CAV1* genotype. By univariate analysis, in the per protocol population, patients receiving a graft carrying *CAV1* rs4730751 AA genotype displayed a significant decrease in estimated glomerular filtration rate (eGFR), whereas eGFR remained stable for patients transplanted with a rs4730751 AC or CC graft (Figure 1). More precisely, eGFR modification between 2 and 5 years post-transplant was −10 ± 9 mL/min/1.73 m^2^ for AA group, 0 ± 11 mL/min/1.73 m^2^ for AC group, and 1 ± 13 mL/min/1.73 m^2^ for CC group (AA *vs* AC, *P* = 0.003; AA *vs* CC, *P* = 0.003) (Figure 2). Two years after transplantation, *CAV1* genotype did not influence recipients’ proteinuria. However, 5 years after transplantation, 35.7% of patients with a *CAV1* AA genotype graft developed proteinuria *vs* 21.3% for AC and 14.3% of patients with CC genotype (*P* < 0.05) (Table 1). In multivariate analysis of the renal transplant function determinants, analysis of covariance for serum creatinine levels (expressed as log10-transformed, Table 2) showed a significant interaction between genotype and time post-transplant (*P* = 0.02) and between genotype and delayed graft function (DGF) (*P* = 0.003). In the group of patients that did not experience DGF, 3-month creatinine levels were similar for all *CAV1* graft genotypes (*P* = 0.10). By contrast, for patients with a past history of DGF (22.1%), 3-month creatinine levels were significantly different (AA *vs* AC: *P* = 0.01; AA *vs* CC: *P* = 0.001; AC *vs* CC: *P* = 0.07). Due to the significant interaction between genotype and time post-transplant, the change in creatinine slopes over time differed significantly according to genotype. Specifically, creatinine increased significantly over time only for genotype AA (*P* = 0.0005). Of note, the slope for genotype AC nearly reached significance (*P* = 0.08). The donor age (*P* < 0.0001) was also an independent predictor of creatinine increase over time (*P* < 0.0001).

**Table 2:** **Covariance analysis of repeated measures for creatinine and estimated glomerular filtration rate**

	**Serum creatinine**			**eGFR (aMDRD)**		
**Variable**	**Beta**	***F***	***P***	**Beta**	***F***	***P***
Intercept	0.9319			1.8358		
*CAV1* genotype		4.93	0.008		5.98	0.003
AA	Reference			Reference		
AC	0.0515			−0.0508		
CC	0.0254			−0.0310		
Time (per year)	0.0262	17.63	<0.0001	−0.0298	18.23	<0.0001
Interaction genotype/time (per year)		3.88	0.02		3.75	0.02
AA	Reference			Reference		
AC	−0.0209			0.0230		
CC	−0.0219			0.0249		
Delayed graft function		0.08	0.78		0.53	0.47
No	Reference			Reference		
Yes	−0.0956			0.1322		
Interaction genotype/DGF		6.02	0.003		6.98	0.001
AA	Reference			Reference		
AC	0.0990			−0.1287		
CC	0.1691			−0.2123		
Donor age (per year)	0.0036	84.10	<0.0001	−0.0045	140.76	<0.0001
Acute rejection			NS		15.15	0.0001
No				Reference		
Yes				−0.0695		

DGF: delayed graft function, eGFR: estimated glomerular filtration rate, aMDRD: abbreviated modification of diet in renal diseases, NS: not significant.

Analysis of covariance for eGFR levels (expressed as log10-transformed) was consistent with the conclusions regarding serum creatinine levels (Table 2). Again, significant interactions were observed between genotype and time (*P* = 0.02) as well as between genotype and delayed graft function (*P* = 0.001). Three-month eGFR levels were not significantly different for patients that did not experience delayed graft function (*P* = 0.24), whereas 3-month eGFR levels were statistically different according to graft genotype for patients that experienced delayed graft function (AA *vs* AC: *P* = 0.01; AA *vs* CC: *P* = 0.0005; AC *vs* CC: *P* = 0.02). In particular, eGFR decreased significantly over time for patients transplanted with a AA graft genotype (*P* = 0.0005) or a AC graft genotype (*P* = 0.04). This decrease was faster for AA genotype compared to AC genotype (test of equality of slopes: *P* = 0.01). Other predictors associated with an eGFR decrease were a higher donor age (*P* < 0.0001) and an acute rejection after transplantation (*P* = 0.0001).

**Figure 3:**
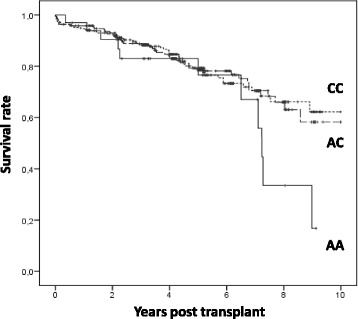
Association between donor *CAV1* rs4730751 Single Nucleotide Polymorphism genotype and death-censored allograft failure.

By Kaplan-Meier analysis, rs4730751 did not affect renal survival (Figure 3).

### Association between rs4730751 and renal graft histology

Regardless of the biopsy indications (systematic or on clinical indication), neither the development of tacrolimus nephrotoxicity nor acute tubular necrosis were influenced by *CAV1* genotype (Table 3). Similarly, the frequency of acute rejection lesions was independent of *CAV1* genotype (data not shown). Concerning the clinically indicated biopsies, the incidence of interstitial fibrotic lesions (ci = 1 and ci = 2) was statistically higher for grafts carrying the rs4730751 AA genotype compared to AC or CC grafts. By contrast, on systematic biopsies, no significant association was found between genotypes and interstitial fibrosis score. Furthermore, as the number of patients exhibiting severe interstitial fibrotic lesions was too low (ci = 3: 6 patients), statistical analysis could not be performed in this subgroup.

**Table 3:** **Impact of** ***CAV1*** **genotype on graft histological lesions**

**Biopsies**	***CAV1*** **genotype**		
	**AA**	**AC**	**CC**
Systematic	*n* = 10	*n* = 43	*n* = 69
Acute tubular necrosis	0	0	1 (1.4%)
Tacrolimus acute tubular toxicity	1 (10.0%)	7 (16.3%)	18 (26.1%)
Tacrolimus chronic vascular toxicity	1 (10.0%)	5 (11.6%)	7 (10.1%)
Acute rejection (cellular or humoral)	0	1 (2.3%)	7 (10.1%)
IF/TA grade I or II	5 (50.0%)	14 (32.6%)	18 (26.1%)
IF/TA grade III	0	1 (2.3%)	1 (1.4%)
Clinically indicated biopsies	*n* = 7	*n* = 38	*n* = 49
Acute tubular necrosis	2 (28.6%)	12 (31.6%)	11 (22.4%)
Tacrolimus acute tubular toxicity	1 (14.3%)	5 (13.2%)	9 (18.4%)
Tacrolimus chronic vascular toxicity	2 (28.6%)	7 (18.4%)	9 (18.4%)
Acute rejection (cellular or humoral)	1 (14.3%)	9 (23.7%)	11 (22.4%)
IF/TA grade I or II	5 (71.4%)^*^	11 (28.9%)^*^	5 (10.2%)^*^
IF/TA grade III	0	1 (2.6%)	3 (6.1%)
Total of biopsies	*n* = 17	*n* = 81	*n* = 118
Acute tubular necrosis	2 (11.8%)	12 (14.8%)	12 (10.2%)
Tacrolimus acute tubular toxicity	3 (17.6%)	12 (14.8%)	27 (22.9%)
Tacrolimus chronic vascular toxicity	3 (17.6%)	12 (14.8%)	16 (13.5%)
Acute rejection (cellular or humoral)	1 (5.9%)	10 (12.3%)	11 (9.3%)
IF/TA grade I or II	10 (58.8%)^**^	25 (30.8%)^**^	23 (19.5%)^**^
IF/TA grade III	0	3 (3.7%)	3 (2.5%)
BK virus nephropathy	2 (11.8%)	3 (3.7%)	4 (3.4%)

^*^ *P* < 0.05. ^**^ *P* < 0.01.

## Discussion

Since the first draft of the human genome has been released, substantial progress has been made in our understanding of the genetic basis of many complex diseases. In particular, large-scale analyses have provided important new insights into the genetic architecture of chronic kidney disease by identifying new susceptibility loci [14,15]. Nevertheless, much less is known about the allelic spectrum for genes underlying kidney fibrosis [16], a progressive pathogenic process ultimately leading to end-stage renal failure. Recently, using a tagging approach, Moore *et al*. identified and validated an association between a common genetic variant (rs4730751) within the *CAV1* gene and renal allograft failure and fibrosis, when present in the donor kidney [4]. Furthermore, CAV1, the primary structural component of plasma membrane caveolae, has independently been identified as a crucial inhibitor of tissue fibrosis and has been functionally implicated in the pathogenesis of various fibrotic disorders including kidney fibrosis [17].

In this study, we investigated whether patients harboring the *CAV1* rs4730751 AA genotype are at higher risk of chronic allograft dysfunction. Our results showed that patients receiving a graft with *CAV1* AA genotype significantly developed more interstitial fibrosis lesions (ci = 1 and ci = 2) and are more prone to experience kidney damages (evaluated by both proteinuria and decrease of glomerular filtration rate) over time. Modification of graft function was observed with both creatinine measurement and estimation of the glomerular filtration rate (using abbreviated modification of diet in renal diseases (aMDRD) formula), suggesting that graft function modification was independent of biases potentially associated to aMDRD formula. Moreover, for patients with a past history of graft-delayed function, CAV1 genotype is a determinant of renal function recovery at 3 months post-transplant. However, no association with tacrolimus-induced lesions of acute or chronic toxicity was found, suggesting that this genotype does not influence tacrolimus nephrotoxicity. Other risk factors associated with worse renal allograft function were donor age and a past history of acute rejection. These risk factors are usually observed in renal transplant cohort studies [18]. The follow-up of our cohort was not sufficient to observe an association between graft survival and CAV1 genotype and thus we were unable to independently replicate findings obtained by Moore *et al*. [4]. Of note, patients receiving a graft with AA genotype associated with the worse outcome were transplanted with younger grafts and experienced delayed graft function to a lesser extent. While non-significant, these observations strengthen the potential impact of donor CAV1 genotype on renal outcome. As donor/recipient mixed chimerism may contribute to kidney fibrosis [19], it cannot be excluded that recipient CAV1 genetic polymorphism may influence graft outcome. Nevertheless, Moore *et al*. failed to find an association between recipient CAV1 rs4730751 and allograft outcome in a large cohort [4].

Although our study, as well as that of Moore *et al*., identified a statistical association between the rs4730751 tag Single Nucleotide Polymorphism (SNP) and renal allograft function, the precise variants that have a causal role remain to be identified [4]. As the rs4730751 tag SNP is not in linkage disequilibrium with other genetic variants located in exons that may truncate or otherwise alter *CAV1* gene product, the causative variant is likely to be regulatory rather than coding. Indeed, for multifactorial traits, most of the genetic variants identified so far have been mapped to non-protein-coding regions, where they influence transcriptional output [20]. Interestingly, Manetti *et al*. recently identified an association between the *CAV1* rs959173 intronic SNP and systemic sclerosis, a connective tissue disease associated with fibrosis [21]. Therefore, *CAV1* genetics is likely to be complex, and deep resequencing at the *CAV1* locus is required to clearly define the causal variants.

## Conclusions

As renal transplantation is suggested as an *in vivo* model of accelerated tissue fibrosis, genotyping of *CAV1* may be relevant in other renal and non-renal diseases characterized by tissue fibrosis. In line with this, Chand *et al*. recently found an association between rs4730751 *CAV1* genetic polymorphism and the prognosis of ANCA associated vasculitis, with a protective effect of the CC genotype [22]. Also, CAV1 genotyping may extend to other chronic kidney disease conditions in which CAV1 is thought to play a major role.

## Methods

### Ethics statement

The protocol has been certified to be in accordance with French laws by the Institutional Review Board of Centre Hospitalier Regional Universitaire de Lille. French health authorities have waived the requirement for consent related to donors who are no longer alive. DNA collection was registered at French ‘Ministère de l’Enseignement Supérieur et de la Recherche’ under the number DC-2008-642. Genotyping analysis and immunosuppressive therapy were performed as described in our local regular protocol of renal transplant care.

### Patients

Four hundred seventy-five French renal recipients consecutively transplanted between 1999 and 2005 participated in this cohort survey. Only recipients of kidney from deceased donor were eligible for inclusion. Patients under 18 years and combined graft recipients were also excluded from this study.

### Immunosuppressive therapy

All patients received initially biological induction (antithymoglobulin (ATG) or anti-CD25 monoclonal antibodies), tacrolimus, Mycophenolate Mofetil (initially 1 g twice daily, thereafter tapered), and steroids (500 mg at day 0, 250 mg at day 1, and next 20 mg/day until day 7). Steroids were stopped at day 8 for patients without immunological risk or delayed graft function. The initial daily dose of tacrolimus (PROGRAF®) was 0.075 mg/kg twice a day (0.15 mg/kg/day). Then, the dose was adjusted to reach a trough blood concentration between 10 and 15 ng/mL the first 3 months, and between 8 and 12 ng/mL within the first year. After 1 year, trough blood levels were targeted between 6 and 8 ng/mL. Nevertheless, the daily tacrolimus dose was adjusted according to the clinical state of the patient.

### Genotyping

Deceased donor DNA was extracted from lymphocytes used for the pre-transplantation cross match test as part of routine practice.

Each amplification reaction was carried out in a total volume of 25 μL 10 mMTris-HCl buffer pH 8.4 containing 50 mM KCl, 0.2 mM of each dNTP, 2 μM MgCl_2_, 0.4 μM of each primer (CAV1F: TGGTATCTAACATACAGCC and CAV1R: GGAGGTATGGCATGTGGA), 200 ng DNA, and 0.6 U Taq DNA polymerase (Life Technologies, Carlsbad, CA, USA). After an initial denaturation step at 94°C for 3 min, 35 cycles of 1 min at 94°C, 1 min of hybridization at 60°C, and 1 min of extension at 72°C were carried out. A final extension period of 7 min was performed at 72°C. Size and specificity of PCR fragments were controlled on 1% agarose gels after incorporation of an intercalator (EvaGreen, Jena Bioscience, Jena, Germany).

After purification with the ExoSap-IT enzyme (USB) (Affymetrix, Santa Clara, CA, USA), amplicon nucleotide sequences were determined using an automated DNA sequencer (ABI Prism® 3130 Genetic Analyser, Life Technologies, Carlsbad, CA, USA). Fragments were amplified with the CAV1F and CAV1R primers, labeled with the BigDye® Terminator v3.1 kit (Life Technologies, Carlsbad, CA, USA) and analyzed with SeqScape v2.5.6 software (Life Technologies, Carlsbad, CA, USA).

### Allograft outcome

During the follow-up period, clinical parameters were recorded, including DGF (defined by the requirement of dialysis session(s) during the first week after transplantation) and the renal function (estimation of the eGFR, according to aMDRD formula [23]. Proteinuria was only recorded at 2 years and 5 years post-transplant and was categorized as followed: no proteinuria (less than 300 mg/24 h or less than 300 mg/g creatinine) or overt proteinuria.

### Histopathology

Retrospectively, histological data were available for the last 207 patients included in this cohort (216 biopsies). One hundred twenty-two biopsies were systematically performed 3 months after transplant, whereas the remaining 94 biopsies were carried out on clinical indication (median time post transplantation: 110 days (88 to 470 days)). Borderline changes were not considered for acute rejection. Pathological criteria for the diagnosis of tacrolimus toxicity were cytoplasmic isometric vacuolization of tubular cells (acute tubular toxicity) and/or arteriolar nodular hyalinosis (chronic arteriolar hyalinosis). The semi-quantitative analysis of graft interstitial fibrosis (ci score) was graded according to the updated Banff 07 classification [24].

### Statistical analysis

To assess the homogeneity of the patient population, genotype frequencies were tested against Hardy-Weinberg equilibrium using a chi-square test.

Categorical variables are described as absolute numbers and proportions, continuous variables as mean ± standard deviation or median [25th to 75th percentiles]. Baseline characteristics according to genotype were compared with Chi-square tests for categorical variables and with analyses of variance for continuous variables.

Serum creatinine data and eGFR data from 3 months to 6 years (2,934 measurements) were analyzed using repeated-measures analysis of covariance (PROC MIXED, SAS Institute, Cary, NC, USA), with time, genotype and their interaction as independent predictors, and adjusted for baseline characteristics. Data were log10-transformed due to skewed distributions. The repeated-measures covariance structure was specified as a spatial power function to handle unequally spaced measurements over time. When either creatinine or eGFR change was not linear with respect to a continuous predictor, the predictor was transformed into a binary variable, the cut-off value being the value minimizing the Bayesian Information Criterion of the model. Parameters of the model were tested with polynomial contrasts. Regression underlying assumptions were visually inspected with residual plots. Statistical significance was set at *α* = 0.05.

Graft survival related to *CAV1* genotype was estimated by the Kaplan-Meier method and compared with the logrank test.

## Abbreviations

ANCA: antineutrophil cytoplasmic antibodies
ATG: antithymoglobulin
BMI: body mass index
CAV1: caveolin 1
DGF: delayed graft function
eGFR: estimated glomerular filtration rate
IF/TA: interstitial fibrosis/tubular atrophy
MDRD: modification of diet in renal diseases
NODAT: new onset diabetes after transplantation
SNP: Single Nucleotide Polymorphism
TGFβ: Transforming growth factor beta
